# Editorial: Flowering time control in agricultural and horticultural crops

**DOI:** 10.3389/fpls.2023.1116197

**Published:** 2023-02-09

**Authors:** Liang Wu, Leo F.M. Marcelis, Fanjiang Kong, Yang Zhu

**Affiliations:** ^1^ Hainan Yazhou Bay Seed Laboratory, Hainan Institute, Zhejiang University, Sanya, Hainan, China; ^2^ College of Agriculture and Biotechnology, Zhejiang University, Hangzhou, Zhejiang, China; ^3^ Horticulture and Product Physiology, Department of Plant Sciences, Wageningen University, Wageningen, Netherlands; ^4^ School of Life Sciences, Guangzhou University, Guangzhou, China

**Keywords:** flowering regulation, photoperiod, vernalization, circadian clock, seasonal flowering, yield potential

Flowering time regulation in higher plants is a crucial developmental phase change controlled by environmental conditions and developmental signaling. The complexity of this regulation divides plant growth into vegetative and reproductive stages, which are processed by an extensive network of floral signaling pathways. Decades of research on flowering time control have discovered several comprehensive systems that plants have evolved to trigger the floral transition. Factors such as day length, temperature, florigen movement, vernalization, and the juvenile phase transition play regulatory roles in flowering control. This Research Topic aims to illustrate advanced findings in flowering time control in agricultural and horticultural plants. Horticultural crops provide special food resources for people, such as fruits, vegetables and tea, and ornamentals add beauty and interest to a garden, thus understanding the nature of their reproductive transitions will reinforce the quality and thereby increasing the income and the prosperity to benefit mankind.

Among the regulatory factors, photoperiod has been considered a central environmental factor for flowering time control in plants. Based on the molecular mechanisms of flowering time, short-day plants (SDPs) and long-day plants (LDPs) have been gradually investigated, which offers an opportunity to explain the changes in day length observed by the two types of plants (Song et al., 2018). In LDP, such as Arabidopsis, CONSTANS (CO) is a pivotal hub integrating numerous internal and external signals for inducing photoperiodic flowering. In SDP, such as rice, the homolog of CO, Hd1, on the other hand, promotes and prevents flowering under SD and LD, respectively (Shim et al., 2017). Many SDPs, such as rice and soybean, have a critical threshold for day length and can even detect changes of 15 minutes for flowering decisions. To better understand the critical day length regulation and the conflict between SDPs and LDPs through photoperiod, Lv et al. conducted a study using next-generation sequencing (NGS)-based bulked segregant analysis (BSA) to map quantitative genes controlling the long-juvenile (LJ) trait in soybean. The LJ trait has been introduced into soybean cultivars to increase yield in tropical environments. The authors identified two genomic regions on scaffold 32 and chromosome 18 harboring loci LJ32 and LJ18, respectively, regulating LJ trait controlling soybean flowering. Their findings will enhance our understanding of the molecular mechanisms underlying the LJ trait and provide useful genetic resources for soybean molecular breeding in tropical regions. In another study, Khan et al. characterized the natural variations in CO family genes and their association with flowering time and maturity in soybean regarding the whole genomic region involving conserved and mutated genes. The authors reported that haplotypes exhibited natural divergence associated with flowering dates and soybean maturity in adapting to diverse environments.

The onset of flowering in plants is precisely controlled by extensive environmental factors and internal molecular networks, in which FLOWERING LOCUS T (FT) is a key flowering integrator (Cho et al., 2017). Yuan et al. studied FT homologs in the soybean genome, diversifying the soybean flowering pathway through characterizing *GmFT3a*, *GmFT2a*, and *GmFT5a*, which are located on the same chromosome as the flowering promoters and act as flowering promoters in the non-inductive photoperiod in soybean. The authors suggested that *GmFT3a* provides an opportunity to slightly promote the flowering time of soybean varieties, which helps retain the yield and agronomic traits of an elite variety with the extension of its adaptive regions (Qi et al., 2021).

The circadian clock and related genes are another internal timing mechanism that allows plants to make decisions in accordance with the environmental conditions. Gong et al. examined the regulatory roles of the circadian clock genes on growth and development of crop species on yield-related traits characterizing CIRCADIAN CLOCK-ASSOCIATED 1 (CCA1) homologue in wheat. Their results provide novel insights into a circadian-mediated mechanism for expressing genes in wheat to coordinate photosynthetic and metabolic activities, leading to optimal growth and development. The authors investigated the tissue-specific expression pattern of TaCCA1 genes in wheat at ZT3 grown under LD conditions. All three TaCCA1 genes exhibited analogous expression patterns with a strong accumulation in green tissues than the other tissues, which were mostly the same as those observed in LHY/CCA1 of Arabidopsis and OsCCA1 of rice (Lu et al., 2009; Sun et al., 2021).

SUPPRESSOR OF OVEREXPRESSION OF CONSTANS1 (SOC1) encodes a MADS-box protein that plays regulatory roles in integrating multiple flowering signals for floral transition and reproductive development in Arabidopsis. Ma and Yan investigated how cotton responds to environmental cues to adjust flowering time in achieving the reproductive success. Cotton is cultivated worldwide due to its broader adaptation to environment and successful breeding for early-matured varieties. This study highlighted the roles of *SOC1* for the integration of endogenous and exogenous signals in plants to maximize reproduction, demonstrating that GhSOC1s may evolve divergently, respond differently to light and temperature, and act cooperatively to promote floral transition in tetraploid cotton.

Transcription factors, besides other endogenous developmental signals, are also involved in the flowering time regulation in plants. WRKY is a large family of transcription factors known for various functions ranging from stress resistance to plant growth and development (Li et al., 2016). Khan et al. studied Chrysanthemum, a well-known ornamental plant with multiple uses, examined the WRKY family and observed a total 138 genes which were classified into III groups. Group III of *C. lavandulifolium* contains 53 members, which is larger than group III of Arabidopsis. AuR and GRE-responsive *cis*-acting elements were located in the promoter region of WRKY members, which are important for plant development and flowering induction. This research provides a basis to study the role of WRKY genes in developing ornamental plants, especially in flowering traits.

Trevaskis et al. wrote a comprehensive review discussing Oat (*Avena sativa*) seasonal flowering behavior as a key contributor in the successful cultivation of oat. Oat is a vernalization-responsive LD plant that flowers after winter as daytime lengthens in spring. Variation in both vernalization and day length requirements broadens the adaptation of oat and has been used for breeding modern cultivars with seasonal flowering behaviors suited to different regions, sowing dates, and farming practices. The authors examined the importance of variation in oat phenology for crop adaptation and outlined strategies to advance our understandings of the genetic basis of oat phenology. Their research emphasized the molecular basis of oat phenology to resolve the contribution of individual genes to crop performance by developing oat genetic resources, such as near-isogenic lines and genetically modified new oat varieties.

Higher and more stable crop yields are the main targets for cereal breeders. It is expected that vernalization requirements of current cultivars can be desynchronized with the environment’s vernalizing potential as the winters in temperate areas will become warmer (Wu et al., 2017). To honor the promise of increased yield potentials, Fernández-Calleja et al. studied hybrid barley phenology deployed in new cultivars. In their study, hybrid combinations extend the available catalog of genetic responses to vernalization, opening new possibilities for optimizing the phenology to specific areas using hybrids. Hybrids can show a more nuanced response to insufficient vernalization than inbred lines proposing new options to manage flowering time based on specific alleles and, particularly, the duration of developmental phases that build yield potential in hybrid barley, suggesting that this strategy could be used in other crops to provide future food security.

RNA sequencing (RNA-Seq) is a powerful tool to examine the continuously changing cellular transcriptome under distinct conditions, therefore, Dong et al. conducted RNA-seq to elucidate the expression of light-related regulatory genes under SD photoperiod inducement of adzuki bean flowering, providing an important theoretical basis for accelerated breeding programs. Their study provides a deep understanding of the molecular mechanisms of adzuki bean flowering in response to SD photoperiod, which laid a foundation for the functional verification of genes delivering an important reference for the molecular breeding of adzuki beans for further studies.

Together, these recently published articles provide a broad overview of the key roles of flowering time control and related genes in agricultural and horticultural crops. This Research Topic highlights innovative and emerging areas in the discipline and opens new routes of discovery that will inspire researchers with extensive research interests.

## Author contributions

All authors listed have made a substantial, direct, and intellectual contribution to the work and approved it for publication.
